# Clinical predictors of microvascular obstruction by delayed enhanced CMR in STEMI patients

**DOI:** 10.1186/1532-429X-13-S1-P143

**Published:** 2011-02-02

**Authors:** José T Ortiz Pérez, María Manuela Izquierdo Gómez, L Bakhos, Ander Regueiro, Teresa María De Caralt, Rosario Jesus Perea, Susana Prat, Daniel C Lee, Xavier Bosch, Edwin Wu

**Affiliations:** 1 Thorax Institute. Hospital Clinic Barcelona, Barcelona, Spain; 2 Feinberg Cardiovascular Institute. Northwestern University, Chicago, IL, USA; 3 Radiology Department. Hospital Clinic Barcelona, Barcelona, Spain

## Objective

To identify significant predictors of the presence and extent of microvascular obstruction in the acute phase of STEMI patients.

## Background

The presence of microvascular obstruction (MO) on delayed enhanced cardiac magnetic resonance (DE-CMR) imaging is associated with adverse remodeling and poor prognosis after STEMI. Identifying which patients that may develop MO prior to undergoing acute mechanical reperfusion maybe important in managing patients for more direct interventions. We sought to evaluate clinical predictors of MO as depicted by DE-CMR.

## Methods

We included 255 patients with their first STEMI reperfused with primary percutaneous intervention. A standard DE-CMR was performed acutely at a mean of 3.9±2.0 days after admission. Clinical risk factors, time to reperfusion as well as angiographic variables were prospectively collected. The angiographic area at risk and the infarct size as a % of the left ventricle (LV) were computed. The number of segments with MO, defined as an area of hypoenhancement surrounded by delayed enhancement on DE-CMR, were summed to calculate MO extent.

## Results

MO was present in 44% of the cases. Patients with MO had 3.1 ± 1.8 segments with MO. Different variables were analyzed but only male gender, diabetes, infarct location anterior, initial TIMI 0/1 flow and absent collaterals (Rentrop’s grades 0/1) were associated with an increased MO incidence as shown in table 1 and had greater number of segments with MO. In addition, greater number of MO segments signifiantly correlated with a larger angiographic area at risk (P=0.005) and infarct size (P<0.001) and borderline with time to reperfusion (P=0.06).

**Table 1:** Mean number of segments with MO

	Yes	No	P value
Male gender	1.5±2.0	0.7±1.5	0.003
Diabetes	1.9±1.9	1.2±1.9	0.009
Anterior Location	1.6±2.2	0.8±1.3	0.004
Initial TIMI flow 0/1	1.5±2.0	0.8±1.4	0.03
Absent Collaterals	1.5±2.1	0.8±1.4	0.02

Important nonsignificant parameters include hypertension, tobacco use, hypercholesterolemia, family history of CAD or final TIMI epicardial flow.

By multivariate regression analysis, the area at risk, male gender, diabetes, absence of collaterals and initial poor TIMI flow remained as the only independent predictors of MO extent.

## Conclusion

The prevalence of MO is often seen in STEMI. Larger MO sizes are seen in diabetics and males, as well as in patients with large areas at risk and poor residual flow prior to mechanical revascularization.

